# The effects of electronic nursing handover on patient safety in the general (non-COVID-19) and COVID-19 intensive care units: a quasi-experimental study

**DOI:** 10.1186/s12913-023-09502-8

**Published:** 2023-05-23

**Authors:** Azadeh Tataei, Bahlol Rahimi, Hadi Lotfnezhad Afshar, Vahid Alinejad, Hossein Jafarizadeh, Naser Parizad

**Affiliations:** 1 Student Research Committee, Urmia University of Medical Sciences, Urmia, Iran; 2 Department of Health Information Technology, School of Allied Medical Sciences, Urmia University of Medical Sciences, Urmia, Iran; 3 Health and Biomedical Informatics Research Center, Urmia University of Medical Sciences, Urmia, Iran; 4 Department of Epidemiology and Biostatistics, School of Medicine, Urmia University of Medical Sciences, Urmia, Iran; 5 Patient Safety Research Center, Clinical Research Institute, Urmia University of Medical Sciences, Urmia, Iran

**Keywords:** Nursing handover, COVID-19, Electronic handover, Patient safety, Intensive care units

## Abstract

**Background:**

The unprecedented increase in the nurses’ workload is one of the issues affecting the quality and safety of patient care in the Intensive Care Units (ICUs). The electronic nursing handover can share sufficient, relevant, and necessary data about patients with greater efficiency and accuracy and prevent their information from being deleted. Therefore, this study aimed to determine and compare the effect of the Electronic Nursing Handover System (ENHS) on patient safety in General ICU and COVID-19 ICU.

**Method:**

This is a quasi-experimental study conducted during an 8-month period from 22 to 2021 to 26 June 2022 using a test-retest design. A total of 29 nurses working in the General and COVID-19 ICUs participated in this study. Data were collected using a five-part questionnaire consisting of demographic information, handover quality, handover efficiency, error reduction, and handover time. Data analysis was conducted in IBM SPSS Statistics for Windows, version 26 (IBM Corp., Armonk, N.Y., USA) using the chi-squared test, paired *t*-test, and Analysis of Covariance (ANCOVA).

**Results:**

The results showed that the mean scores of handover quality and efficiency, reduction of clinical error, and handover time in the electronic handover were significantly higher than those obtained in the paper-based method. The results showed that the mean score of patient safety in the COVID-19 ICU was 177.40 ± 30.416 for the paper-based handover and 251.40 ± 29.049 for the electronic handover (p = .0001). Moreover, the mean score of patient safety in the general ICU was 209.21 ± 23.072 for the paper-based handover and 251.93 ± 23.381 for the electronic one (*p* = .0001).

**Conclusion:**

The use of ENHS significantly improved the quality and efficiency of shift handover, reduced the possibility of clinical error, saved handover time, and finally increased patient safety compared to the paper-based method. The results also showed the positive perspectives of ICU nurses toward the positive effect of ENHS on the patient safety improvement.

**Supplementary Information:**

The online version contains supplementary material available at 10.1186/s12913-023-09502-8.

## Introduction

Patient safety is known as one of the basic components of health systems and a global concern as well. The provision and maintenance of patient safety increase the probability of success in achieving desired treatment results and this is one of the most important challenges for healthcare providers [1]. One of the criteria for ensuring patient safety is to pay attention to “correct nursing handover”, so that an inappropriate nursing handover is one of the most important causes of harm to the patient [2]. Clinical handover is usually conducted using face-to-face verbal communication, structured documented information, medical records, and electronic systems [3, 4]. The verbal and paper-based handover is one of the most common methods of clinical handover, which can cause significant clinical errors and inefficiencies. In the paper-based handover method, relying on memory to transfer patients’ clinical data is not safe enough and handwritten notes resulting from this method may be illegible or incomplete. Moreover, completing paper checklists may increase the handover time. It should be also noted that paper-based clinical handovers cannot be easily examined and evaluated [5]. Accurate clinical handover is of great importance for the continuity and safety of care. If relevant clinical information is not shared accurately and punctually, it may lead to adverse events, delay in treatment and diagnosis, inappropriate treatment, and omission of care [6]. Nekoei-Moghadam et al. (2020) pointed out that the first step to reduce medical errors is to improve patient safety culture in healthcare organizations, so developing health information systems, encouraging employees to report errors, and planning to reduce nurses’ workload can be effective in achieving desired health outcomes. They also found that the level of patient safety in Iran is at a moderate level [7]. The use of new technologies, especially web and mobile applications, is useful for documenting performance, reporting clinical handovers, and standardizing the communication process between nurses and other healthcare professionals [8]. It has also been proven that Electronic Nursing Handover Systems (ENHSs) are more efficient compared to paper-based handover methods and are shown to cause better continuity of care in medical centers. Based on the results of several studies conducted about the development of ENHSs, it has been indicated that these systems provide accurate information for having a structured handover, increase the quality of clinical information, reduce handover time, and ultimately improve communication and users satisfaction as well [9, 10]. Ryan et al. (2011) also showed that the introduction of a new handover system was associated with a significant reduction in patient length of stay in emergency departments and also improved the continuity of patient care [11]. Regarding the complexity of the clinical conditions and the treatment process, the presence of many electronic equipment, patients’ loss of consciousness, and their dependence on others, Intensive Care Units (ICUs) are one of the leading wards causing unintentional harm to patients [12]. Health professionals have applied monitoring technologies for decades in ICUs [13]. The significant increase in critically ill patients throughout the COVID-19 pandemic has affected the workload of ICU nurses as well as the optimal management of the nurse-patient ratio to ensure the quality and safety of patient care [14, 15]. On the other hand, since the subject of patient safety is of great importance in ICUs, especially the COVID-19 ICU [16], it seems absolutely necessary to investigate the effect of ENHS in such departments. In Iran, several studies have been conducted on nurses’ attitudes toward communication skills, barriers to effective communication, handover audits, and patient safety culture [17–19]. However, there has been no study conducted regarding the development and design of an electronic system to improve the nursing handover process. So, we believed that electronizing the nursing handover could affect patient safety. Therefore, this study aimed to investigate ICU nurses’ perspectives toward the effect of ENHS on patient safety and the factors involved in it.

## Materials & methods

### Study design

This is a quasi-experimental study conducted using a pretest-posttest design to evaluate nurses’ perspectives toward ENHS and its effect on patient safety. Quasi-experimental studies are defined as forms of experimental research used to establish a cause and/or effect of an intervention on a population without randomization [20].

This study was conducted during a 10-month period from 23 to 2021 to 22 August 2022 in the 400-bed Imam Khomeini University hospital, Urmia, Iran. The study population consisted of nurses working in the two departments of the COVID-19 and General ICUs. The General ICU provided care for non-COVID patients). The sampling was conducted from 16 April to 1 August 2022.

### Design of electronic nursing handover system

#### Literature review

To design the prototype of ENHS, we were required to find patients’ minimum data set transferred by nurses during the nursing handover. Accordingly, we first carefully studied the related literature about the development of ENHSs and extracted the necessary data in this regard as much as possible.

#### Interview

In the next step, to ensure the completion of the minimum set of data required for the system development as well as its localization, we conducted individual interviews with 12 highly experienced supervisors and nurses, during which the aim of the electronic system development was first explained to them, and their comments were then received. The interviews were recorded and transcribed, and we finally developed the prototype of ENHS in Microsoft PowerPoint format based on the data obtained. Next, we presented the system to a number of five professors and experts in nursing and medical informatics to ensure its accuracy, completeness, and acceptability both in terms of content and appearance. Based on the final expert evaluation, we applied the supplementary items and prepared the final version of ENHS for the encoding stage.

#### Development & validity of ENHS

The ENHS was written in C-Sharp (C#) programming language using the framework of ASP.NET Core MVC and ASP.NET Core Razor with back-end development and the framework of Bootstrap with front-end development on the web in a responsive manner. Ajax technology was also used to create dynamic pages and quickly update a part of the page, which is not possible in classic web pages. Moreover, SQL Server was applied to save data. For the online use of all nurses, a host was created that supported the framework of ASP.NET Core 6.0 and SQL Server 2019 database. The ENHS included items on patients’ demographic information, medical records, vital signs, medical tests, consultations, para clinical measures, medications, and the specialized information assessed in the ICU including pain score, Glasgow Coma Scale (GCS), Full Outline of Unresponsiveness (FOUR) score, etc. It was tried to make the system comprehensible and user-friendly by entering information into it using a selective and optional method so that there was no need to type any long nursing reports (free-text).

#### Intervention

The pilot application of the system was conducted in the two General (non-COVID) and COVID-19 ICUs. We used the census sampling method in this study. Out of 34 nurses working in the two units, 29 entered the study with their informed consent. Each head nurse was individually taught in two sections. Then, they gave their permission to create training groups on WhatsApp messenger, in which basic explanations about the system and study objectives were presented to the participant nurses. Moreover, the necessary data on the group allocation and the time of face-to-face training sessions were announced to them. The participants were provided with 45-minute face-to-face training sessions in 9 groups of 3 people. Moreover, a supplemental training video was also embedded to ensure the completion of training at the beginning of logging into the system. Individual usernames and passwords were defined for all the nurses, which helped to maintain the confidentiality of the patients’ information. After the completion of training sessions, nurses in each unit used the ENHS along with the conventional paper-based handover method for two months. During the ENHS application, the research team had direct supervision to clear any ambiguity in applying the system.

### Data collection

Two researcher-made questionnaires were developed to evaluate nurses’ views regarding paper-based and electronic handovers. The items of the questionnaires were created using the analysis of similar scientific literature and interviews with the nursing team in a centralized group. Then these items were classified into 4 dimensions. The questionnaires included five sections including demographic information, handover quality, handover efficiency, handover errors, and handover time. Nurses were asked to estimate whether they agree or disagree with the set of statements for each of the four defined safety dimensions regarding the paper-based and electronic handover methods based on a 5-point Likert scale. Negative-wording questions were scored reversely so that higher scores indicated a more negative view toward the system. The questions of each dimension were tried to be carefully selected. In general, the questions mainly include issues such as the quality of information transferred between nurses, the conditions for providing better nursing care, the optimization of the handover process, the time spent for handover and the exact time of conducting it, and the errors occurred in data entry or transfer. Two questionnaires included identical and non-identical items. For instance, there were particular items in the ENHS-related questionnaire that questioned the software capability of the system. Nonetheless, most of the questions were designed similarly in a parallel way.

To increase the likelihood that the questionnaires would serve their purpose, their face validity was assessed by gathering feedback provided by an expert panel, which was made up of six experts in medical informatics, nursing, and statistics. The above showed that there was a strong agreement between different groups of experts and this indicated a good understanding of face validity in the questionnaire. In the pretest stage conducted with the coordination of the relevant experts, redundant items were eliminated without removing any dimension in the questionnaire. The pilot test of the questionnaire was conducted by presenting it to 10 ICU nurses and asking them to complete it with a focus on the clarity, relevance, and arrangement of the items based on the dimensions. They also evaluated the content validity of the questionnaire, based on which its CVI was obtained to be 0.85 and 0.87 before and after the intervention, respectively. The reliability was examined by calculating Cronbach’s α coefficient, which was 0.893 and 0.969 before and after the intervention, respectively.

The first questionnaire was completed by the nurses in the two ICUs and then collected two months before the introduction of the system to evaluate their perspectives regarding conventional (paper-based) nursing handover. The pilot test of the system lasted for two months in the two ICUs. Then the same nurses completed the second questionnaire.

### Data analysis

Data analysis was conducted using IBM SPSS Statistics for Windows, version 26 (IBM Corp., Armonk, N.Y., USA). Statistical significance was considered to be less than 0.05. The data were reported as “mean ± standard error.“ Chi-squared test was used to check the frequency distribution of demographic variables. According to the results of the Kolmogorov-Smirnov test, all variables of handover quality, handover efficiency, error reduction, and handover time were found to be normal before and after the intervention. Regarding the normality of all variables, the paired-sample t-test was applied to compare the mean scores of the variables before and after the intervention. Besides, the Analysis of Covariance (ANCOVA) was used to control the confounding effect to compare the mean scores between the two units.

## Results

The demographic characteristics of the participants are shown in Table 1. The demographic information of the respondents was presented in Table 1. A total of 58 questionnaires were collected in this study by 29 nurses (29 questionnaires in pretest and 29 questionnaires in posttest). Of the 29 nurses and supervisors who participated in this study, 25 (86.20%) were female and 4 (13.79%) were male. The nurses were mostly in the 20–40 age group and their mean age was 32.58 years. Moreover, 93.3% of the participants had a bachelor’s degree. The cumulative frequency distribution of age and work experience was not normal between the general and COVID-19 ICUs (*p* = .015 for the general ICU, *p* = .027 for the COVID-19 ICU). However, variables of gender and education had similar cumulative frequency distributions between the two groups. Therefore, age and work experience were identified as confounders. In short, the nurses working in the COVID-19 ICU were partially younger, and the work experience of nurses working in the general ICU was noticeably higher.

**Table 1:** Cumulative Frequency distribution of demographic variables

Variables		Department				*p*-value^*^
		COVID-19 ICU		General ICU		
		Frequency	Percent	Frequency	Percent	
Age	21–30	10	66.7	2	14.3	0.015
	31–40	4	26.7	8	57.1	
	41–50	1	6.7	4	28.6	
Gender	Female	11	73.3	14	100	0.057
	Male	4	26.7	0	0	
Work experience	0–5	8	53.3	1	7.1	0.027
	6–10	3	20.0	6	42.9	
	10<	4	26.7	7	50.0	
Education	Bachelor’s degree	14	93.3	13	92.9	0.741
	Master’s degree	1	6.7	1	7.1	

* Chi-squared test

**Table 2:** Comparison of the mean scores of patient safety in the General and COVID-19 ICUs

Department	Paper-based shift handover	Electronic shift handover	*p*-value^*^
COVID-19 ICU	177.40 ± 30.416	29.049 ± 251.40	0.001
General ICU	23.072 ± 209.21	23.381 ± 251.93	0.001

* Independent-samples *t*-test

Based on the results of the Kolmogorov-Smirnov test, the distribution of the mean score of patient safety was found to be normal before and after the intervention. The results showed that the mean score of patient safety in the COVID-19 ICU was 177.40 ± 30.416 for the paper-based shift handover and 251.40 ± 29.049 for the electronic shift handover (Table 2). This difference in the mean score of patient safety was statistically significant in the COVID-19 ICU (*p* = .0001). Moreover, the mean score of patient safety in the general ICU was 209.21 ± 23.072 for the paper-based shift handover and 251.93 ± 23.381 for the electronic one. The difference in the mean score of patient safety was also statistically significant in the general ICU (*p* = .0001). It was also shown that there was a significant increase in the mean score of positive answers in both units after working with the ENHS. This result can indicate the positive view of nurses toward the use of ENHS for improving patient safety in ICUs.

**Table 3:** Comparison of the mean score of patient safety dimensions in the General and the COVID-19 ICU

Department	Patient safety dimensions	Shift handover method		*p*-value^*^
		Paper-based shift handover	Electronic shift handover	
General ICU	Handover quality	76.14 ± 9.937	92.64 ± 9.927	0.001
	Handover efficiency	57.00 ± 6.961	69.64 ± 6.172	0.001
	Error reduction	55.93 ± 5.704	63.71 ± 6.474	0.05
	Handover time	20.14 ± 3.255	25.93 ± 2.999	0.001
COVID-19 ICU	Handover quality	64.73 ± 10.694	91.40 ± 11.012	0.0001
	Handover efficiency	49.60 ± 9.257	68.93 ± 10.138	0.001
	Error reduction	45.73 ± 8.084	65.67 ± 7.007	0.001
	Handover time	17.33 ± 4.287	25.40 ± 3.089	0.001

* Paired-samples *t*-test

The mean score of handover quality in the general ICU was 76.14 ± 9.937 for the paper-based shift handover and 92.64 ± 9.927 for the electronic shift handover (*p* = .001). The mean score of handover efficiency was 57.00 ± 6.961 for the paper-based shift handover and 69.64 ± 6.172 for electronic shift handovers in the general ICU (*p* = .001). The mean score of error reduction in the general ICU was 55.93 ± 5.704 for the paper-based shift handover and 63.71 ± 6.474 for the electronic one (*p* = .05). The mean score of handover time in the general ICU was obtained to be 20.14 ± 3.255 for the paper-based shift handover and 25.93 ± 2.999 for the electronic one (*p* = .001). The mean score of handover quality in the COVID-19 ICU was shown to be 64.73 ± 10.694 for the paper-based shift handover and 91.40 ± 11.01 for the electronic shift handover (*p* = .0001). The mean score of handover efficiency in the COVID-19 ICU was indicated to be 49.60 ± 9.257 for the paper-based shift handover and 68.93 ± 10.138 for the electronic shift handover (*p* = .001). The mean score of error reduction in the COVID-19 ICU was 45.73 ± 8.084 for the paper-based shift handover and 65.67 ± 7.007 for the electronic one (*p* = .05). The mean score of handover time in the COVID-19 ICU was obtained to be 17.33 ± 4.287 for the paper-based shift handover and 25.40 ± 3.089 for the electronic one (*p* = .001). The difference in the mean scores of all the above variables was statistically significant and the use of ENHS had a positive effect on the quality and efficiency of handover, error reduction, and handover time from the points of view of nurses working in the two ICUs (Table 3).

**Table 4:** Comparison of the mean scores of patient safety dimensions before the use of ENHS in terms of department

	Patient safety dimensions	Department		*p*-value^*^
		COVID-19 ICU	General ICU	
Paper-based shift handover	Handover quality	64.73 ± 10.694	76.14 ± 9.937	0.006
	Handover efficiency	49.60 ± 9.257	57.00 ± 6.961	0.022
	Error reduction	45.73 ± 8.084	55.93 ± 5.704	0.001
	Handover time	17.33 ± 4.287	20.14 ± 3.255	0.057

* Independent-samples *t*-test

The mean score of handover quality was 64.73 ± 10.694 for the COVID-19 ICU and 76.14 ± 9.937 for the general ICU (*p* = .006). Furthermore, the mean score of handover efficiency was 49.60 ± 9.257 for the COVID-19 ICU and 57.00 ± 6.961 for the General ICU (*p* = .022). The mean score of error reduction was 8.084 ± 45.73 for the COVID-19 ICU and 5.704 ± 55.93 for the general ICU (*p* = .022). Based on the results of the independent-samples *t*-test, the mean scores of the above dimensions were not similar between the two units before the intervention in the paper-based shift handover, and nurses working in the general ICU had a more positive perspective towards the provision of patient safety in paper-based shift handover compared to those working in the COVID-19 ICU. However, the result showed that the mean scores of handover time were almost the same between the two units in the paper-based shift handover (*p* = .057) (Table 4).

**Table 5:** Comparison of the mean scores of patient safety dimensions between the two units after the use of ENHS

	Patient safety dimensions	Department		*p*-value^*^
		COVID-19 ICU	General ICU	
Electronic shift handover	Handover quality	91.40 ± 11.012	92.64 ± 9.927	F = 0.072 P = 0.790
	Handover efficiency	68.93 ± 10.138	69.64 ± 6.172	F = 0.004 P = 0.952
	Error reduction	65.67 ± 7.007	63.71 ± 6.474	F = 0.554 P = 0.464
	Handover time	25.40 ± 3.089	25.93 ± 2.999	F = 0.172 P = 0.681

* Analysis of Covariance (ANCOVA)

Based on the results of ANCOVA provided in Table 5, the mean score of handover quality was obtained to be 91.40 ± 11.01 for the COVID-19 ICU and 92.64 ± 9.927 for the general ICU. After controlling the confounding effects of the mean score of handover quality, work experience, and age, the difference in the mean score of handover quality was indicated to be not statistically significant between the two ICUs (*p* = .790). Moreover, the mean score of handover efficiency was shown to be 68.93 ± 10.138 for the COVID-19 ICU and 69.64 ± 6.172 for the general ICU. After controlling the confounding effects of the mean score of handover efficiency in the paper-based handover, work experience, and age, the difference in the handover efficiency was found to be not statistically significant between the two ICUs (*p* = .952). The mean score of error reduction was indicated to be 65.67 ± 7.007 for the COVID-19 ICU and 63.71 ± 6.474 for the general ICU. After controlling the confounding effects of work experience and age, the difference in the mean score of error reduction was found to be not statistically significant between the two ICUs (*p* = .464). The mean score of handover time was obtained to be 25.40 ± 3.089 for the COVID-19 ICU and 25.93 ± 2.999 for the general ICU. After controlling the confounding effects of work experience and age, the difference was found to be not statistically significant (*p* = .681). All of the above results indicated that the ENHS was able to function positively and equally in both the COVID-19 and general ICUs.

**Fig. 1:**
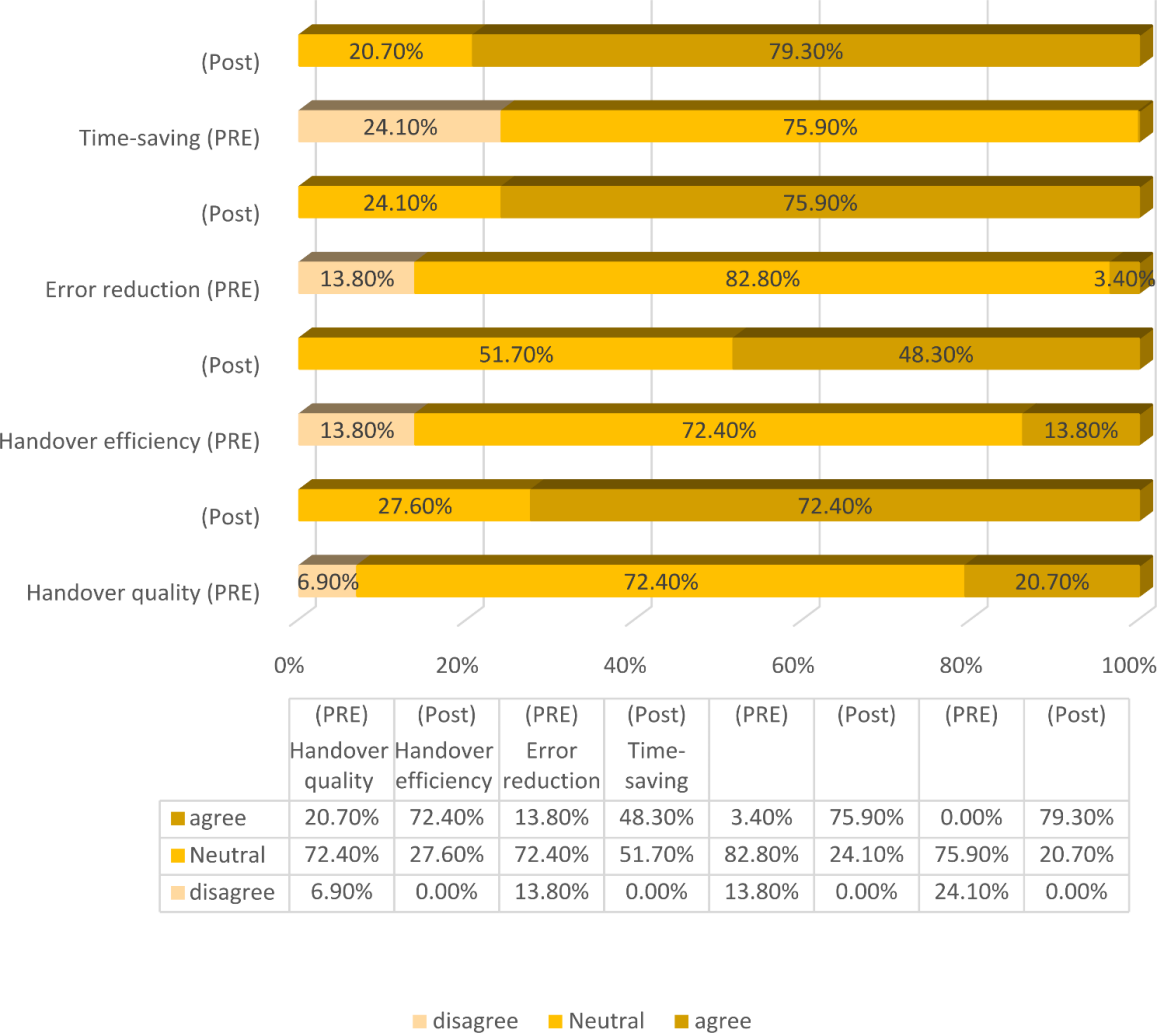
Comparison of ICU nurses’ perceptions of the ENHS before and after the intervention

Based on the standard created in Fig. 1, two responses including “Strongly Disagree,“ and “Disagree” were categorized in the group of opponents, the response of “I have no idea” was considered in the group of neutrals, and those including “Strongly Agree” and “Agree” were allocated to the group of supporters. The results demonstrated that all measured dimensions (outcomes) improved after using ENHS. The percentage of responses agreeing with the improvement of handover quality increased from 20.70 to 72.40%. Besides, 48.30% of nurses agreed that using ENHS improved the handover efficacy compared to the conventional method. Moreover, a significant increase was observed in the percentage of positive responses from 3.40 to 75.90% for error reduction and from 0.00 to 79.30% for handover time.

## Discussion

The present study was conducted to determine and compare the effect of ENHS on patient safety in the general and COVID-19 ICUs. During the COVID-19 pandemic, significant investment has been made in digital technology to support eHealth solutions and provide new ways of delivering services. Nowadays, EHSs are increasingly being used to provide an efficient and standardized platform for information exchange [21]. We used the existing conditions to develop and test the ENHS. As a forward-looking project, the ENHS focused on improving the clarity of transferring clinical data of ICU patients and ultimately increasing patient safety. This study also aimed to sustainably improve the handover quality and efficiency, reduce possible errors and save time during nursing handovers.

Given that handover is an important aspect of providing safe patient care, we prioritized safety as a key issue and used technology to deliver consistent interventions and gain nurses’ confidence in the ENHS. Based on the opinions presented by nurses after the intervention, it was indicated that the ENHS improved the quality of nursing handover and provided a higher level of care for ICU nurses, especially during the COVID-19 pandemic, when the increase in nursing workload has challenged the quality of shift handovers [22]. A high nursing workload may reduce the quality of patient care and threaten patient safety [23]. Thomson et al. (2018) also acknowledged that poor quality or ineffective shift handover could lead to negative outcomes for patients, and the use of technology such as electronic documentation, bedside documentation devices, and other care technologies has the potential to improve the quality of handover by organizing and simplifying the provision of patient’s clinical information [24]. Of course, we should not ignore the fact that the evaluation in our study was conducted by people who were actively part of it (participants), which can be a reason for a positive evaluation of the process.

It can be inferred from the results that using electronic nursing Kardex can improve the quality of handover and clinical efficiency. Since our handover system was developed based on the information needs of nurses during nursing handover, it had a user-oriented environment that was accessible and easy to use for all nurses. Several studies have also pointed out that users’ professional needs should be placed at the core of the system implementation process when developing a clinical computer or informatics system that users interact with in their daily activities. All of the above requires the users’ feedback on the systems to be continuously collected [16, 25, 26]. In line with the results of our study, Jacob et al. (2021) also showed that the efficiency of medical ward rounds can be improved by developing an EHS for surgical ward staff, which can be uploaded and run on iPads during the course. In fact, the use of an iPad for handover after surgery made it possible to access the key clinical information of patients from all parts of the hospital. Accordingly, all procedures were performed on time, effective use of time was possible, and clinical efficiency ultimately enhanced [27].

The results of the present study showed that the use of ENHS, in addition to reducing the handover time, could eliminate data repetition by updating the clinical information at the handover time and, accordingly, reduce the possibility of medical errors. Sun et al. (2018) developed an EHS integrated with EHR for the medical-surgical department and could reduce the shift handover time from 10.5 to 4.4 min. They also claimed that relying on memory and handwritten notes taken from verbal handovers that may be illegible or incomplete is dangerous and increases the handover time [28]. All of the above was also correct for nurses who participated in our study. Since the nurse-to-patient ratio is unreasonably high in Iran, nurses do not have enough time to entirely and accurately transfer clinical information every shift. Kim et al. (2014) also pointed out that these conditions can raise the possibility of irrelevant and incomplete handover and deletion of information as well [29]. Skelton et al. (2019) conducted a study that aimed to reduce the number of medical errors outside office hours by ensuring continuous and clear weekend shift handover through a user-friendly and powerful EHS that was added to the existing information technology system. The findings of their study showed that the formalization and integration of out-of-office hours handover processes in the patient’s electronic record could help reduce errors, increase clarity, and provide better patient care [30]. Lee et al. (2019) conducted a study on nurses’ perceptions and experiences during the transition to an electronic handover informatics system in hospitals. In contrast with the findings of our study, the perspectives of their nurse participants regarding such a system indicated that the dual handover method (paper-electronic) might cause more workload on the employees and delay the acceptance of the new method. Furthermore, having too much information on a web page, providing duplicate information, and ignoring people’s real conditions may also lead to increased errors during shift handover. However, nurses’ opinions regarding the reduction of nursing handover time were consistent with the results of our study [31].

Nowadays, the high importance of patient safety has caused different studies to be conducted in this regard. Pun (2023) developed and evaluated a simulation-based approach to enhance structured and interactive nursing clinical handover using bilingual ISBAR and CARE-team protocols to improve patient safety and continuity of care for nurses. The language used to develop the approach was Chinese. After conducting the simulation-based training, nurses reported that they carried out the clinical handover with more confidence and that this approach led them to have more structured and interactive clinical handovers. Overall, it was indicated that nurses’ communication significantly improved during the handover [32].

## Limitations

According to a survey conducted among ICU nurses, we observed significant improvements in patient safety in the ICU. Despite the above, several limitations existed in this study. Due to the short period of ENHS pilot application, the study could not accurately predict long-term compliance. Moreover, the data were obtained from a limited number of nurses, which might cause biased results. In this study, a pretest-posttest design was utilized, and this method may be confounded by response shift bias since people traditionally improve after the study period for various reasons (e.g., intending to be nice to the researchers). This may confound the observed improvement rate.

Regarding the large number of patients hospitalized in the ICU and nurses’ heavy workload during the COVID-19 pandemic, the ENHS efficiency may not have been fully realized. Accordingly, researchers and program developers are recommended to develop and test an EHS over a more extended time and in a broader study population. To evaluate the current process of nursing shift handover, another study should be organized so as not to be affected by the confounding factors of the pretest-posttest design.

## Conclusion

The ENHS significantly improves handover quality and efficiency, reduces the possibility of medical error, and saves handover time compared to conventional (paper-based) shift handover. The results of our study showed the positive perspectives of nurses towards the positive effect of ENHS on improving patient safety. The research team recommends using the ENHS and comparing it with other handover technologies in future studies.

## Electronic supplementary material

Below is the link to the electronic supplementary material.

Supplementary Material 1

## Data Availability

All data generated or analyzed during this study are included in this article.

## Abbreviations

ENHS: Electronic Nursing handover system
IT: Information Technology
ICU: Intensive Care Unit
EHS: Electronic Handover System
CDC: Centers for Disease Control
