# Supplementary material for: The effects of electronic nursing handover on patient safety in the general (non-COVID-19) and COVID-19 intensive care units: a quasi-experimental study
Source: BMC Health Serv Res. 2023 May 23;23:527. doi: 10.1186/s12913-023-09502-8 (PMC10204677; doi:10.1186/s12913-023-09502-8)
Supplement: Supplementary file 1 — Supplementary Material 1 [file 12913_2023_9502_MOESM1_ESM.pdf]

The questionnaire you are looking at is about the evaluation of paper-based handover, which actually includes two parts: 1- Demographic information 2- The main questions related to the evaluation of the paper-based handover method. Therefore, your cooperation in accurately answering these questions creates the conditions for achieving appropriate results.Thanks

1- Demographic information

Gender: Female ☐ Man ☐

Age: 20-30 ☐ 31-40 ☐ 41-50 ☐ 51-60 ☐

Level of Education: Diploma ☐ Bachelor's degree ☐ Master's degree ☐ PhD ☐

Department: COVID-19 ICUs ☐ General ICUs ☐

Work Experience: 0-5 ☐ 6-10 ☐ >10 ☐

2-Main questions

Handover quality	Strongly Disagree	Disagree	No Idea	Agree	Strongly Agree
1. Paper-based shift handover conveys enough information.					
2. Paper-based shift handover conveys information related to the patient.					
3. Paper-based shift handover conveys essential information.					
4. Paper-based shift handover provides acceptable and valid information.					
5. In the Paper-based shift handover, information is transferred in an organized manner					
6. Paper-based shift handover provides valid documents.					

7. Paper-based shift handover enables clear and transparent information transmission.					
8. The information is transmitted incompletely in Paper-based shift handover					
9. Information is conveyed in an unclear and unintelligible way in paper-based shift delivery.					
10. Paper-based shift handover is easy					
11. Paper-based shift handover provides a high level of security for the patient.					
12. Paper-based shift handover is reliable.					
13. Paper-based shift handover has increased the readability of the data.					
14. The Paper-based shift handover keeps patients' medical information confidential.					
15. Paper-based shift handover provides complete documentation in paper form.					
16. The Paper-based shift handover transmits patients' information accurately.					

17. The Paper-based shift handover reduces inconsistent information recording.					
18. I felt comfortable enough using the Paper-based shift handover.					
19. Using the Paper-based shift handover makes nurses behave more safely.					
20. Paper-based shift handover follows a logical structure.					
21. Paper-based shift handover Provides a common understanding of policies related to patient safety.					
**Handover efficiency**					
1- I get distracted in Paper-based shift handover.					
2- I have enough concentration in Paper-based shift handover.					
3- Paper-based shift handover causes a lot of stress to the nurses.					
4- Paper-based shift handover brings a lot of fatigue for nurses.					
5- Paper-based shift handover cuases a lot of pressure on nurses .					

6- Paper-based shift handover also provides access to medication changes or deletions.					
7- Paper-based shift handover also provides public access to the drug list.					
8- Paper-based shift handover provides an overview of previous and current drug doses for patients.					
9- Paper-based shift handover is done quickly.					
10- The Paper-based shift handover helps to identify the change of position of patients.					
11- The Paper-based shift handover guarantees a professional work report.					
12- Paper-based shift handover promotes patient participation in care.					
13- Paper-based shift handover prevents delays in patient care.					
14- Paper-based shift handover informs the nurses about the patient's condition.					

15- The Paper-based shift handover improves the recall of the information provided.					
16- In the Paper-based shift handover of the shift, the examination of the treatment process of the patients is facilitated by the nurses.					
17- Shift delivery can be tracked on paper.					
**Error reduction**					
1- Paper-based shift handover prevents patient information from being deleted.					
2- Paper-based shift handover avoids irrelevant clinical discussion.					
3- In Paper-based shift handover, medication errors are reduced.					
4- In Paper-based shift handover itself leads to medication errors.					
5- Paper-based shift handover leads to more side effects.					
6- Paper-based shift handover requires additional documentation.					
7- Paper-based shift handover reduces the risk of error.					

8- Paper-based shift handover makes it possible to correct the error.					
9- Paper-based shift handover reduces incorrect information.					
10- Paper-based shift handover prevents documentation errors.					
11- Paper-based shift handover reduces the number of mistakes in data transfer.					
12- Paper-based shift handover reduces the error related to shift delivery.					
13- Paper-based shift handover reduces errors not related to shift delivery.					
14- In Paper-based shift handover, the reporting error is significantly reduced.					
15- Paper-based shift handover , patient safety problems that have occurred many times are ignored.					
16- In Paper-based shift handover, important patient care information is often lost.					
**Time-saving**					
1- Paper-based shift handover takes a long time.					

2- Paper-based shift handover saves time.					
3- By using Paper-based shift handover, the nursing report prepared in a reasonable time.					
4- Paper-based shift handover Done in a reasonable time.					
5- information is transferred on Time in Paper-based shift handover					
6- The Paper-based shift handover reduces work interruptions.					
